# Collection and Lipidomic Analysis of Murine Knee Synovium and Infrapatellar Fat Pad

**DOI:** 10.3390/mps9030070

**Published:** 2026-05-02

**Authors:** Tong Yang, Luke Stasikelis, Alexander J. Knights

**Affiliations:** 1 Department of Orthopaedic Surgery, Washington University, St. Louis, MO 63110, USA; 2 Center of Regenerative Medicine, Washington University, St. Louis, MO 63110, USA

**Keywords:** osteoarthritis, synovium, infrapatellar fat pad, mass spectrometry, lipidomics

## Abstract

Intra-articular soft connective tissues such as synovium and adipose tissue play a crucial role in governing joint homeostasis and disease progression in various forms of arthritis. In the knee, like many synovial joints, adipose tissue forms an integrated anatomic and functional unit with the joint-lining synovium, and the most prominent adipose depot is the infrapatellar fat pad (IFP). With growing evidence that lipid profiles in the synovium–IFP unit shift during progression of joint diseases like osteoarthritis (OA), there is strong impetus for consistent tissue collection approaches and reproducible subsequent lipid characterization. Here, we present a standardized dissection and low-input untargeted lipidomics workflow optimized for mouse knee synovium and IFP, to enable comprehensive lipid profiling. Synovium/IFP from multiple joints are pooled to increase input mass and guarantee robust lipid yield, followed by lipid extraction and high-resolution liquid chromatography-mass spectrometry (LC–MS) acquisition for global, untargeted lipidomic profiling. The analysis workflow encompasses robust feature detection, accurate lipid annotation, data transformation and normalization. These steps enhance comparability across samples, particularly those with low input amounts, while minimizing technical variance and batch effects. Using this approach, we detect a broad spectrum of lipid species spanning the major lipid categories. As expected for untargeted discovery, a subset of non-lipid species is also observed. This protocol provides a practical framework for robust, reproducible lipidomics in murine intra-articular soft tissues to support future disease-specific biomarker and drug target discovery in OA and other joint diseases.

## 1. Introduction

Arthritis is a term that broadly encompasses joint diseases which exhibit coordinated pathological changes in cartilage, subchondral bone, synovium, joint fat, and other articular tissues [1]. In a knee-specific context, the synovium that lines the joint forms a functionally and anatomically integrated unit with intra-articular adipose tissue, in which immune cells, fibroblasts, and adipocytes engage in dynamic crosstalk to regulate homeostasis, but can also promote initiation and progression of diseases like OA [2,3]. Several adipose depots exist within the knee joint, but the most prominent and best studied is the infrapatellar fat pad (IFP), also called ‘Hoffa’s fat pad’, after the German orthopedic surgeon Albert Hoffa, who first described this structure. The synovium–IFP unit is well positioned to regulate joint lipid homeostasis—IFP adipocytes provide a lipid-rich depot, while synovium is a highly active immune niche [4]. Together, these integrated tissues form an immunometabolic microenvironment poised to both generate and respond to bioactive lipid networks that, if dysregulated, can shape disease onset and progression [5,6].

Lipids represent a highly diverse and chemically heterogeneous class of biomolecules that are essential for virtually all aspects of cellular and organismal function. They encompass a broad array of structures, with fundamental roles in energy storage and metabolism, cellular structure, biosynthesis, and signaling. ‘Lipidomics’ refers to the comprehensive analysis of lipid species, their abundances, and dynamic changes in biological systems. Over the past decade, accumulating mass spectrometry–based lipidomic studies in OA, for instance, have revealed that lipid categories in the joint shift across disease stages [7,8]. Recent studies further show that lipid pathways intersect with key hallmarks of disease, including synovitis and pain signaling [9,10], suggesting a role for lipid dysregulation as both a mechanistic driver and a source of translational biomarkers. Collectively, these findings underscore the value of unbiased, untargeted lipidomic approaches to comprehensively profile joint lipids and to pinpoint disease-specific lipid pathways and actionable targets linked to complex, multi-tissue diseases like OA and other types of arthritis.

Murine models of arthritis are widely used in pre-clinical research for many reasons—they facilitate mechanistic dissection of pathways that are difficult to address in heterogeneous human cohorts [11,12], and allow for control of experimental variables such as age, sex, genotype, injury timing, etc. However, murine intra-articular tissues yield extremely limited material due to their minute size and anatomic delicacy, plus, are prone to unwanted tissue contamination during dissection [13]. Given the sensitivity of mass spectrometry-based lipidomic approaches, when input is inherently limited, even small differences in sample collection and processing can disproportionately influence measurements, distort interpretation, and compromise cross-study reproducibility [14,15].

To address these challenges, we developed a detailed, step-by-step, standardized workflow optimized for mouse knee synovium and IFP collection, followed by performance of untargeted lipidomics and analysis. This protocol is designed to reduce pre-analytical variation, maximize inter-operator consistency, and facilitate cross-study comparability for joint lipidomics studies focused on joint homeostasis and disease.

## 2. Experimental Design

**Figure 1:**
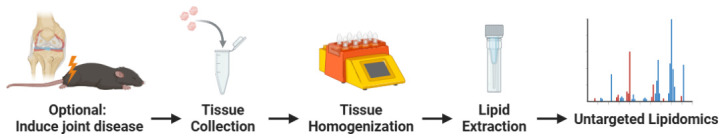
Schematic overview of tissue collection, processing, and downstream lipidomics. Images were created with Biorender.com.

The workflow comprises five stages (Figure 1):

(i) Optional: induction of joint disease model in mice;
(ii) Harvest of knee synovium and IFP;
(iii) Tissue homogenization;
(iv) Lipid extraction;
(v) LC–MS acquisition and data analysis.

This workflow can be adapted to any pre-clinical model of joint disease, or can be performed using healthy mice, as done here. As proof of concept, here we used naïve C57BL/6J mice to obtain tissues, and all procedures performed were approved by our Institutional Animal Care and Use Committee (IACUC).

The tissue collection process was optimized for small intra-articular tissues such as synovium and IFP, with careful dissection to minimize contamination from adjacent muscle or tendon, thereby ensuring tissue specificity for downstream analysis. Our prior single-cell RNA-sequencing data validated that this dissection protocol yields only very minimal contamination from surrounding skeletal muscle, constituting <1% of total cells [16]. Due to the limited size of individual mouse knee tissues, synovium and IFP from 2 to 3 knee joints are typically pooled to generate a single sample for lipidomics. This pooling approach ensures sufficient tissue mass for robust extraction and detection, especially when using an untargeted approach.

Thorough homogenization ensures efficient lipid recovery from small-volume tissues, thereby maximizing yield despite the limited starting material. The biphasic methanol–methyl tert-butyl ether (MTBE) lipid extraction method is employed to achieve broad category coverage, with particular effectiveness for relatively polar lipid species. High-resolution LC–MS analysis is performed in data-dependent acquisition mode to maximize lipid coverage and facilitate structural identification via MS/MS fragmentation.

Due to the extremely limited amount of synovium and IFP tissue obtained from mouse knees joints, total lipid yield per sample is constrained. As a result, pooled quality control (QC) samples were omitted from the workflow to preserve biological material. Instead, technical variability was mitigated through mathematical normalization and statistical modeling strategies, including normalization to tissue weight, Log_2_ transformation, and probabilistic quotient normalization (PQN)-based normalization. Differential lipid abundance was assessed using empirical Bayes-based linear models, incorporating paired designs when comparing contralateral knees from the same mouse.

### 2.1. Materials

- Dry ice;
- 1.5 mL collection tubes;
- Dissection mat;
- 70% Ethanol (*v*/*v*) in spray bottle;
- Methyl tert-butyl ether (MTBE), LC–MS grade (Sigma-Aldrich, St. Louis, MO, USA);
- Methanol (MeOH), LC–MS grade (Fisher Scientific, Waltham, MA, USA);
- Acetonitrile, LC–MS grade (Fisher Scientific, Waltham, MA, USA);
- Water, LC–MS grade (Fisher Scientific, Waltham, MA, USA);
- gentleMACS™ M Tubes (Miltenyi Biotec, Bergisch Gladbach, Germany).

### 2.2. Equipment

- Dissection stand with alligator clamps, magnifier, light source;
- Medium scissors (Roboz, Gaithersburg, MD, USA; RS-6700);
- Two medium serrated forceps (Roboz, Gaithersburg, MD, USA; RS-8100);
- Two scalpel handles, #3 (Roboz, Gaithersburg, MD, USA; RS-9843);
- #11 scalpel blades (Fisher Scientific, Waltham, MA, USA);
- Microscissors (Roboz, Gaithersburg, MD, USA; RS-5600);
- Fine forceps (Roboz, Gaithersburg, MD, USA; RS-5110);
- Precision Balance (Mettler Toledo, Columbus, OH, USA; ME303E);
- gentleMACS™ Dissociator (Miltenyi Biotec, Auburn, CA, USA);
- Probe sonicator (Fisher Scientific, Waltham, MA, USA; FB120);
- Refrigerated microcentrifuge (Eppendorf, Hamburg, Germany; 5425R);
- SpeedVac vacuum concentrator (Thermo Fisher Scientific, Waltham, MA, USA);
- Vanquish Horizon UHPLC system (Thermo Fisher Scientific, Waltham, MA, USA);
- BEH C8 column, 2.1 mm × 100 mm, 1.7 µm (Waters Corporation, Milford, MA, USA);
- High-resolution Orbitrap ID-X Tribrid mass spectrometer (Thermo Fisher Scientific, San Jose, CA, USA).

## 3. Procedure

All steps should be performed in accordance with institutional animal care standards and biosafety regulations. Use appropriate personal protective equipment and handle sharps and organic solvents with care and dispose of waste appropriately.

### 3.1. Pre-Dissection Preparation (10–15 min)

Prepare collection tubes: Place pre-labeled 1.5 mL microcentrifuge tubes on dry ice in advance. These will be used to immediately collect and snap-freeze tissue.

Set up dissection area: Arrange a clean dissection mat under a stereomicroscope or magnifier with adequate lighting. Lay out all dissection tools (scissors, forceps, scalpels, etc.) for easy access. Ensure the work area is sanitized (e.g., wipe down with 70% ethanol).

Tool sterilization (optional): For lipidomics workflows, absolute sterility is not required, but cleanliness is important to avoid contamination (e.g., residual grease or plastics can introduce lipid contaminants).

### 3.2. Harvest of Knee Synovium and Infrapatellar Fat Pad (15 min per Knee)

**Figure 2:**
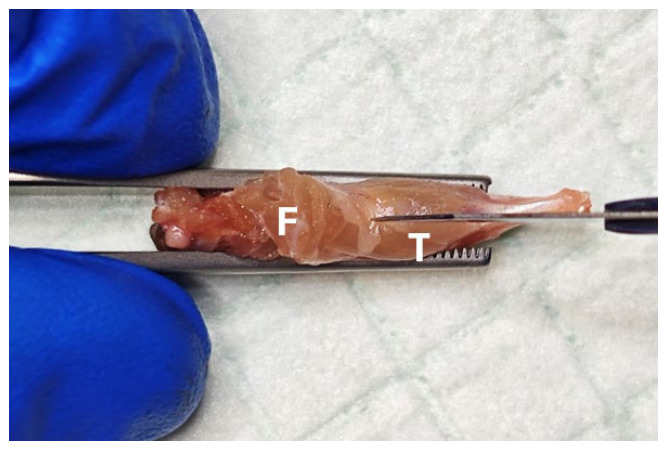
Preparatory cut to flay posterior muscles. F, femur; T, tibia.

**Figure 3:**
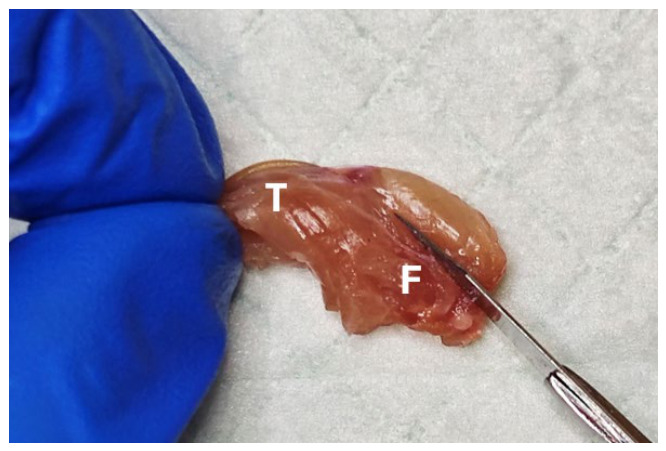
Preparatory cut to detach quadriceps from femur. F, femur; T, tibia.

**Figure 4:**
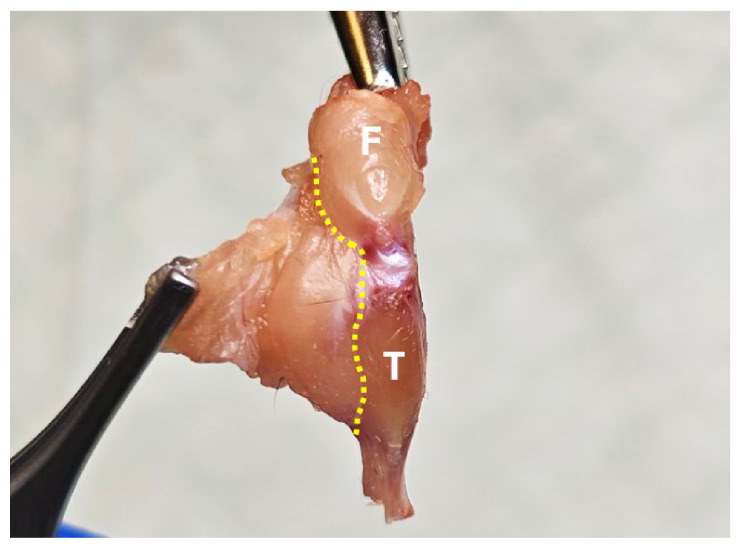
Trim off the posterior muscles along the yellow dashed line. F, femur; T, tibia.

**Figure 5:**
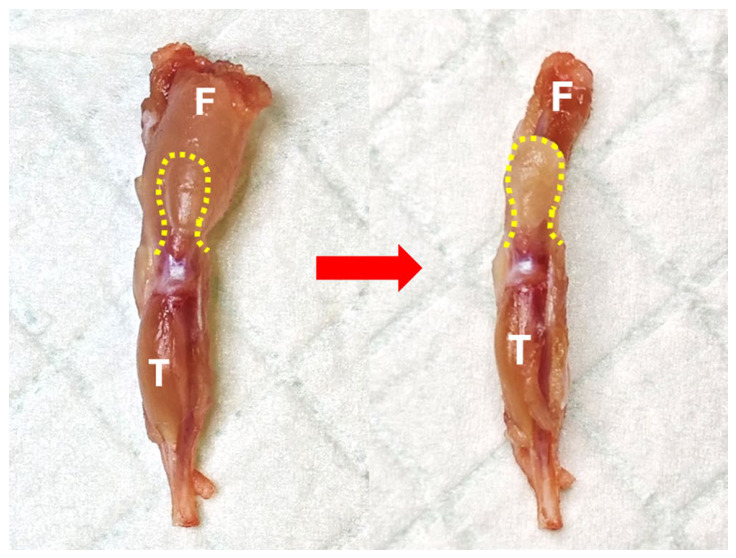
Trim off the bulk quadriceps muscle along the yellow dashed line. (**Left**), before trimming; (**right**), after trimming. F, femur; T, tibia.

**Figure 6:**
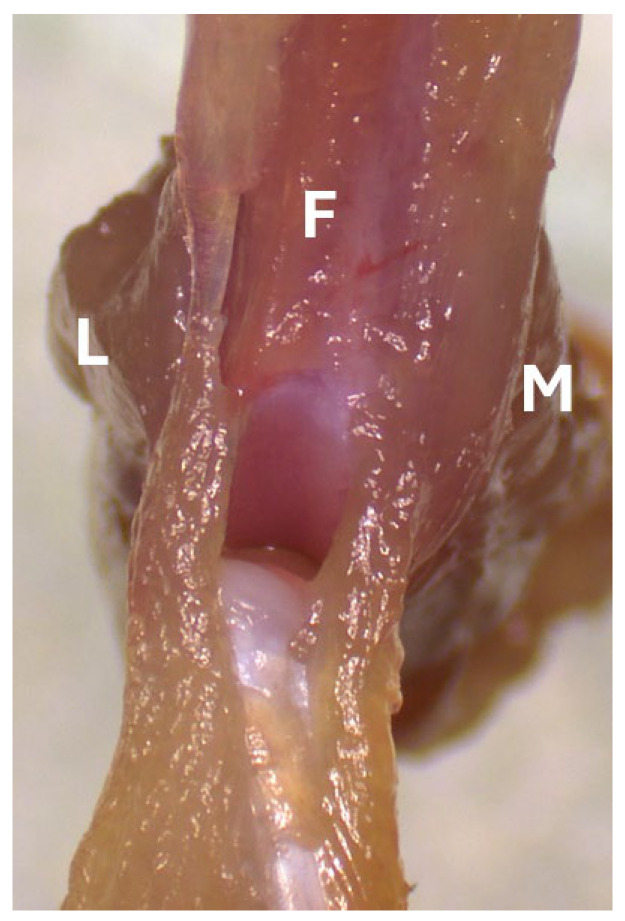
Gently retract the remaining quadriceps to expose the knee joint space. F, femur; L, lateral side; M, medial side.

**Figure 7:**
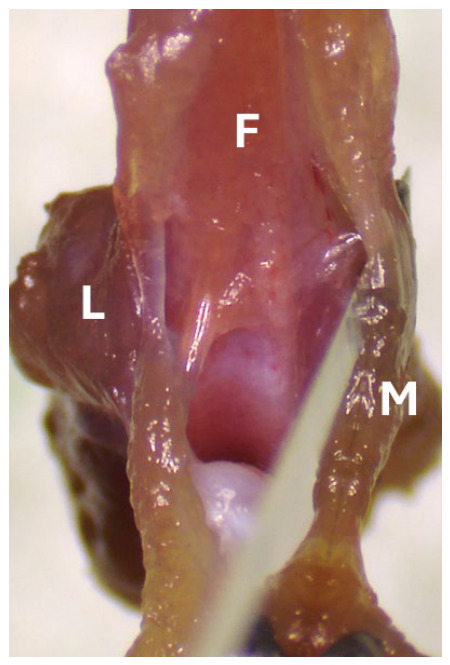
Use a scalpel to cut the synovium along the medial femoral condyle. F, femur; L, lateral side; M, medial side.

1. Euthanasia: Euthanize the mouse using an approved method based on institutional animal care guidelines. Spray the hindlimb with 70% ethanol to dampen fur and reduce potential contamination during dissection.
2. Limb preparation: Make a transverse incision across the dorsal lower back and reflect the skin to expose the pelvis and hindlimbs. Disarticulate the femoral head at the hip joint and remove the entire hindlimb. Remove the paw at the ankle joint before proceeding with knee dissection.
3. Preparatory cuts for muscle trimming:

- Position the removed limb securely between forceps, with the posterior side facing upwards. Using a scalpel, make a longitudinal incision from the superior aspect of tibia to the ankle, flaying the distal hamstring and calf muscles open to each side of the knee and lower limb (Figure 2).
- Rotate the limb onto its side. Beginning just above the knee joint, separate the quadriceps from the femur proximally toward the femoral head (Figure 3), while keeping the quadriceps tendon attached to the patella and avoiding disruption of the joint capsule.

4. Remove posterior muscle: Clamping the femoral head for support, use serrated forceps to pull the flayed calf and hamstring muscles laterally away from the joint. Trim gross muscle as well as any muscle adhering to the joint capsule using scissors, then repeat on opposing side of the joint (Figure 4). Be careful not to cut into the joint capsule—a small amount of residual muscle is acceptable and can be removed at a later step.

5. Remove bulk quadriceps muscle: Make a small incision toward the quadriceps tendon to allow for removal of gross medial and lateral portions of quadriceps muscle while preserving a small part of the central portion attached to the quadriceps tendon (Figure 5). Maintain the tendon–patella connection to enable traction during subsequent steps.

6. Open the joint capsule:

- Gently retract the remaining quadriceps with forceps to create tension and allow access inside the joint space (Figure 6). Use a new blade before proceeding, to avoid contaminating intra-articular tissues with extra-articular tissue residue.
- Shallowly insert a #11 scalpel blade into the joint cavity just posterior to the patella. Rotate the blade medially and cut along the inner surface of the joint capsule (synovial lining) at the femoral condyles, staying as close to the femur as possible (Figure 7). Extend the cut as far posteriorly as possible to maximize medial synovium yield while avoiding transection of the tissue.
- Repeat the cut on the opposite (lateral) side. Gently pull the remaining quadriceps distally to fully expose the joint capsule.

Note: Using this approach, a greater amount of synovial tissue is typically recovered from the lateral side of the knee joint.

**Figure 8:**
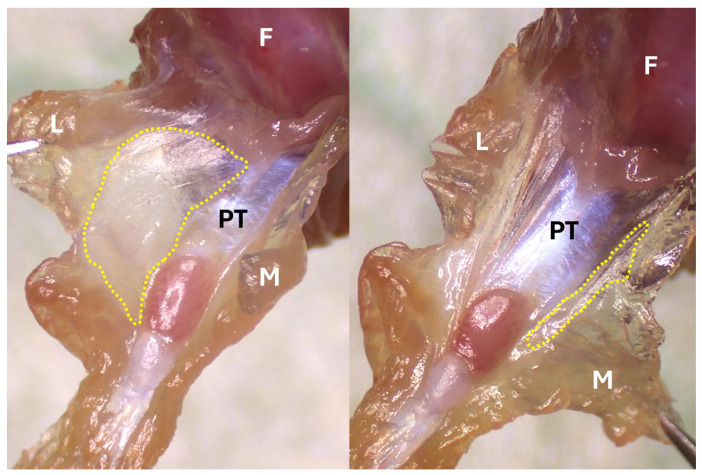
Lateral (**left**) and medial (**right**) synovium outlined with yellow dashed line. F, femur; L, lateral side; M, medial side; PT, patellar tendon.

**Figure 9:**
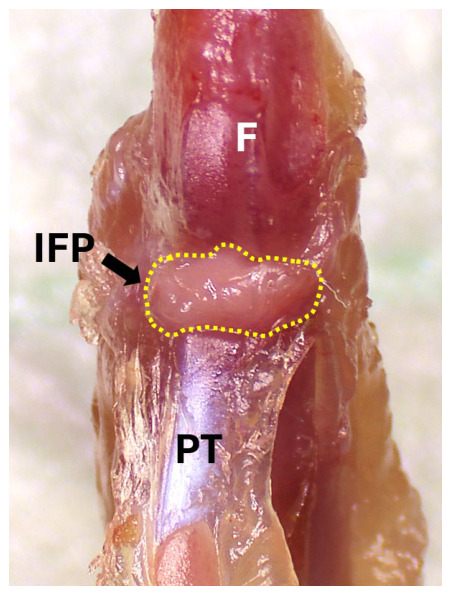
Identify the IFP within the knee joint; the IFP is outlined with yellow dashed line. F, femur; IFP, infrapatellar fat pad; PT, patellar tendon.

7. Collect the synovium: Use micro forceps to pull the joint capsule away from the joint, to fully expose the lateral and medial synovium (Figure 8). Use micro scissors and trim any residual muscle surrounding the synovium on the medial and lateral sides. After all muscle surrounding the synovium has been removed, using micro forceps, grasp the synovial tissue around the patellar tendon (which appears as a translucent membrane, distinct from reddish surrounding muscle) and carefully cut it off along the border between synovium and the opaque white patellar tendon. Note that it is very difficult to cleanly harvest posterior synovium tissue, so it is not included in this workflow.

8. Collect the infrapatellar fat pad (IFP): Identify the IFP as a yellowish fat mass located just inferior to the patella and deep to the patellar tendon (Figure 9). Use a scalpel to gently disassociate the fat pad from the joint cavity. With fine forceps, pull the fat pad away while cutting its attachments with micro scissors or a scalpel. Transfer the IFP tissue into the same tube containing the synovium (if a combined analysis is intended). Note that the IFP will inherently contain anterior synovium tissue, since these two tissues are closely integrated.

Critical steps:

1. Consistency of dissection boundaries is essential for reducing variance in any downstream analysis. When multiple operators are involved, conduct side-by-side training and periodic cross-checks to ensure the harvested tissue regions and typical tissue yields are consistent across users.
2. Avoid including excess non-synovial tissue (muscle, tendon, cartilage) in the sample, as this can dilute tissue specific lipid signals and introduce lipids not originating from the synovium or IFP. Careful dissection and visual confirmation of tissue identity help to ensure sample purity.

### 3.3. Snap-Freezing and Storage (5 min)

Immediately snap-freeze the tube containing the collected synovium and IFP tissue by placing it directly onto dry ice. Rapid freezing preserves the lipid composition by preventing post-mortem degradation.

Store the frozen tissue samples at −80 °C until lipid extraction. It is recommended to proceed to extraction as soon as feasible. Avoid freeze–thaw cycles, as repeated thawing can alter the lipid profile.

### 3.4. Lipid Extraction (Overnight)

1. Weigh each frozen synovium and infrapatellar fat pad tissue sample. Record tissue wet weight for downstream data normalization.
2. Add methanol to each sample and homogenize using gentleMACS™ M Tubes with a gentleMACS Dissociator using gentleMACS Program RNA_01.
3. Transfer the homogenized lysate to a new 1.5 mL microcentrifuge tube using a wide-bore pipette tip.
4. Sonicate the lysate using a probe sonicator (3 cycles at 30% amplitude; 30 s on, 55 s off) to fully disrupt any residual tissue fragments.
5. Dry the sonicated lysate completely using a SpeedVac concentrator with no heat until all visible liquid is evaporated.
6. Add 1 mL methanol/MTBE mixture (3:10, *v*/*v*) to each tube containing dried residue.
7. Vortex vigorously for 1 min to resuspend lipids and promote extraction.
8. Incubate the samples at −80 °C overnight to enhance lipid recovery and protein precipitation.
9. Add 300 μL LC-MS grade water to induce biphasic separation.
10. Centrifuge at 12,000× *g* for 10 min at 4 °C to separate layers.
11. Carefully collect the upper organic phase (MTBE-rich) containing lipids, without disturbing the interphase.
12. Add fresh MTBE and water to the remaining aqueous phase (same volumes as above), vortex vigorously for 1 min, centrifuge at 12,000× *g* for 10 min at 4 °C, and collect the second upper phase.
13. Combine both organic phases in a clean tube and dry completely in a SpeedVac concentrator (room temperature, no heat).
14. Reconstitute the dried lipid film with a mixture of methanol, acetonitrile, and water (50:25:25, *v*/*v*/*v*, typically 30 µL). Vortex to fully dissolve.

### 3.5. Liquid Chromatography-Mass Spectrometry Acquisition (20 min per Sample)

1. Perform liquid chromatography using a Waters BEH C8 column (2.1 × 100 mm, 1.7 µm, Waters) with a 16 min gradient.
2. Use mobile phase A: 10 mM ammonium acetate with 5% methanol and 0.1% acetic acid in water, and mobile phase B: 0.1% acetic acid in methanol.
3. Set the flow rate to 0.4 mL/min and maintain the column temperature at 50 °C.
4. Inject 5 µL of each reconstituted sample into a Vanquish Horizon UHPLC system (Thermo Fisher).
5. Analyze eluted lipids using an Orbitrap ID-X Tribrid mass spectrometer (Thermo Fisher) equipped with an electrospray ionization source.
6. Acquire data in both positive and negative ion modes using AcquireX DeepScan for untargeted lipidomics. Collect MS1 and MS2 scans at 60,000 resolution over an *m*/*z* range of 200–1100. Set capillary voltages to 3.4 kV (positive mode) and 2.6 kV (negative mode); set ion transfer tube temperature to 325 °C.

### 3.6. Data Processing and Statistical Analysis (1–2 h)

1. Feature extraction and identification: Process raw LC–MS files in Compound Discoverer (v3.4; Thermo Fisher Scientific) for peak detection and retention time alignment. Perform MS/MS-based annotations by spectral matching against mzVault, mzCloud, LipidBlast, and LipidSearch.
2. Quality filtering: Retain features detected in at least 50% of samples within all experimental groups, and remove obvious artifacts/poorly integrated peaks during manual curation.
3. Normalization to sample input: Normalize lipid intensities to wet tissue weight to account for variable tissue yield. This step improves quantitative comparability when tissue yield varies across dissections.
4. Data transformation: Log_2_-transform weight-normalized intensities to stabilize variance and improve approximate normality across lipid features prior to statistical modeling.
5. Mathematical normalization to reduce technical variability: Apply probabilistic quotient normalization (PQN) after log_2_ transformation to mitigate sample-to-sample intensity scaling differences.
6. Differential abundance testing: Assess differential lipid abundance between groups using the limma framework (linear modeling with empirical Bayes variance moderation), which provides more stable variance estimates than feature-wise *t*-tests in small-sample settings. For within-mouse comparisons, implement a paired design by including mouse ID as a blocking factor in limma.
7. Multiple testing and reporting: Given the modest number of curated lipid features, users can prioritize biological interpretation by reporting effect sizes (log_2_ fold change) alongside both nominal and False discovery rate (FDR)-adjusted *p*-values. Nominal *p*-values (typically *p* < 0.05) are used to flag suggestive changes for hypothesis generation, while FDR < 0.05 is used to define statistically robust differences.

## 4. Expected Results

Using the standardized dissection workflow described here, synovium and IFP collected and pooled from three healthy mouse knee joints are expected to yield a combined wet weight of 5–8 mg. Careful separation along anatomical boundaries should minimize carryover from surrounding tissues, resulting in samples with low muscle contamination and more consistent tissue composition across operators and cohorts. It is worth noting that the appearance and amount of synovium and IFP tissue may vary dramatically depending on the healthy or diseased status of the animal—and as such, yields may also vary.

**Figure 10:**
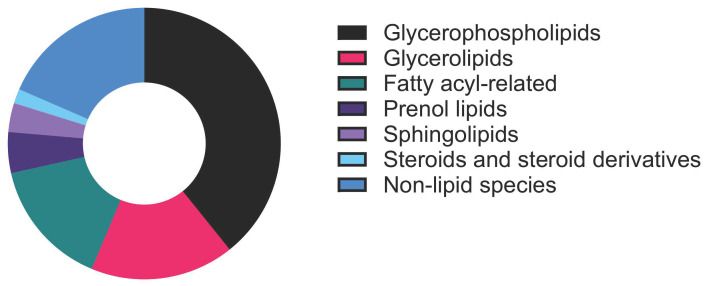
Pie chart showing the distribution of lipid and non-lipid species detected by untargeted lipidomics of healthy mouse knee joint synovium and IFP.

The stated tissue pooling approach is expected to provide sufficient input for untargeted LC–MS analysis, enabling robust detection of a broad spectrum of lipid species with stable signal intensity across major lipid categories (Figure 10).

Glycerophospholipids and glycerolipids should comprise a large fraction of the detected features (Figure 10 and Table 1), both in terms of number of species and overall signal, consistent with their high baseline abundance in tissues [17]. Fatty acyls and sphingolipids are also expected to be reliably detected, but typically represent fewer species and lower overall intensity. In addition to lipids, we anticipate detection of a subset of non-lipid species (Figure 10 and Table 2), which is expected for untargeted acquisition, as the lipid extraction and LC-MS conditions capture a broad range of small molecules beyond lipids that co-elute or ionize efficiently under the same workflow, and database-driven feature annotation can assign high-confidence hits to non-lipid metabolites [18]. Overall, LC–MS acquisition of tissue from three pooled knees should provide comprehensive lipid category coverage while also revealing predictable non-lipid features inherent to metabolomics-style profiling.

**Figure 11:**
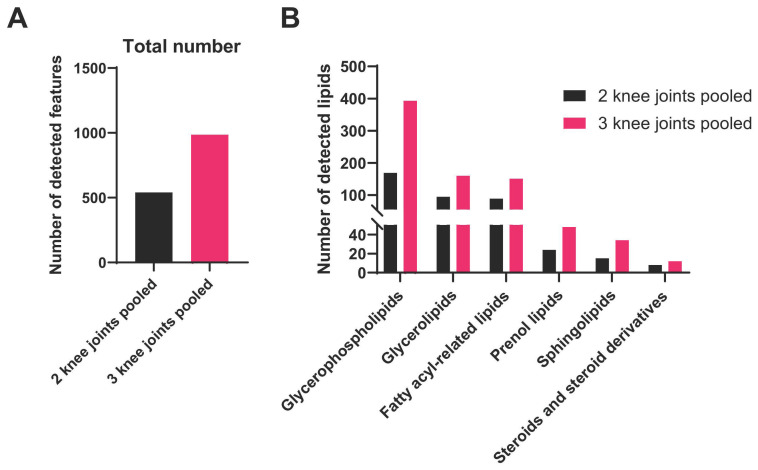
Total number of detected features and category-level lipid composition in untargeted LC–MS, comparing tissue pooling from two versus three pooled knee joints. Shown are (**A**) the total number of detected species (lipid + non-lipid) and (**B**) the number of detected lipid species within each major lipid category.

To define a practical minimum input and illustrate the benefit of increased tissue mass, we compared pooling tissues from two versus three knee joints. Using the same lipid extraction and LC–MS acquisition workflow, pooling three knee joints increased overall feature detection (Figure 11A) and consistently improved coverage across all major lipid categories (Figure 11B). This trend was observed across categories, with three-joint pools yielding more detected species than two-joint pools in each lipid category. This threshold provides an empirical benchmark for experimental planning and cross-study reproducibility. Overall, a relatively comprehensive lipidomic profile should be obtained from murine synovium–IFP samples. If a specific lipid class is not detected or is consistently underrepresented, one possible cause is suboptimal extraction or recovery of that lipid species. In such cases, adding an appropriate class-matched internal standard during the lipid extraction step can help verify extraction efficiency and improve confidence that the method adequately captures the lipid class of interest.

In this workflow, untargeted lipidomics provides broad coverage of lipid features and enables discovery of unanticipated lipid changes, making it well suited for unbiased hypothesis generation. However, untargeted approaches typically yield relative rather than absolute quantification and are often less sensitive for low-abundance compounds than targeted methods. By contrast, targeted lipidomics offers higher sensitivity and specificity and enables more accurate absolute quantification, but its narrower scope—limited to a predefined panel—reduces scalability for discovering new biomarkers [19]. Ideally, untargeted profiling is used to nominate candidate lipids and pathways, which are then validated and quantified with targeted assays for key lipid species.

We anticipate that this workflow will yield a high-confidence feature result with accurate *m*/*z*, retention time, and MS/MS-supported annotations suitable for downstream statistical testing. To improve comparability across low-input synovium/IFP samples and reduce technical variance, feature intensities should be Log_2_-transformed and normalized using PQN, a robust math-based approach that corrects for sample-amount/dilution effects in untargeted LC–MS metabolomics-style datasets [20,21]. Given the extremely limited yield of murine intra-articular tissues, we prioritize allocating material to biological samples rather than routine pooled quality control injections; however, when feasible, inclusion of pooled quality control samples is widely recommended to strengthen analytical robustness and cross-study reproducibility in untargeted MS workflows [22]. Users may also consider incorporating a surrogate quality control sample for monitoring analytical stability, without using valuable experimental samples.

This protocol is limited by the inherently low yield of the murine synovium–IFP unit, which can restrict downstream analyses given the limited sample input. In addition, the dissection procedure described does not capture the posterior synovium, and so the collected tissue may not fully represent the entire synovial compartment. Because these tissues are very small, reproducible processing may also require dedicated homogenization equipment to ensure efficient tissue disruption and extraction.

Overall, this workflow provides a practical and scalable lipidomics framework for studying synovium and IFP, and their role in joint homeostasis and diseases like OA. It enables robust and reproducible detection of disease-associated lipid remodeling across murine intra-articular soft tissues and supports future mechanistic links between lipid pathways and joint disease progression.

## Figures and Tables

**Table 1:** Major lipid categories detected by untargeted lipidomics.

Major Lipid Categories	Sub-Classes	Total Number Detected
Glycerophospholipids	PC, PE, PI, PS, PG, PA	387
Glycerolipids	TG, DG, MG	168
Fatty acyl-related lipids	Fatty acids and conjugates, fatty alcohols/ester, fatty amides, eicosanoids	150
Prenol lipids	Isoprenoids, quinones, terpenoids	48
Sphingolipids	Cer, SM, glycosphingolipids	34
Steroids and steroid derivatives	Steroids, hydroxysteroids, steroid esters/lactones	17

**Table 2:** Non-lipid species detected by untargeted lipidomics.

Non-Lipid Species	Sub-Classes	Total Number Detected
Small oxygenated/sulfur/phosphate-containing organics	Alcohol and polyols; ethers; carbonyl compounds; phosphate esters; sulfuric acid esters	48
Nitrogen-containing metabolites	Amino acids, peptides, and analogs; amines; piperazines	24
Organic acids and derivatives	Benzoic acids and derivatives; medium-chain hydroxy acids and derivatives; beta hydroxy acids and derivatives; tricarboxylic acids and derivatives	25
Aromatic/polyphenolic compounds	Diphenylmethanes; phenylpropanes; isoflav-2-enes; tetrahydrofuran lignans	16
Carbohydrates and carbohydrate conjugates	Carbohydrates and carbohydrate conjugates	6

## Data Availability

The raw data supporting the conclusions of this article will be made available by the authors on request.

## Abbreviations

The following abbreviations are used in this manuscript:

OA	Osteoarthritis
IFP	Infrapatellar fat pad
LC-MS	Liquid chromatography–mass spectrometry
MS/MS	Tandem mass spectrometry
MTBE	Methyl tert-butyl ether
PA	Phosphatidic acid
PC	Phosphatidylcholine
PE	Phosphatidylethanolamine
PG	Phosphatidylglycerol
PI	Phosphatidylinositol
PS	Phosphatidylserine
MG	Monoacylglycerol
DG	Diacylglycerol
TG	Triacylglycerol
Cer	Ceramide
SM	Sphingomyelin
